# Fixed night workers and failed smoking cessation

**DOI:** 10.1186/s12995-019-0243-z

**Published:** 2019-08-05

**Authors:** Youn-Mo Cho, Hyoung-Ryoul Kim, Mo-Yeol Kang, Jun-Pyo Myong, Jung Wan Koo

**Affiliations:** Department of Occupational & Environmental Medicine, College of Medicine, The Catholic University of Korea, 222, Banpo-daero, Seocho-gu, Seoul, Seoul Republic of Korea

**Keywords:** Smoke cessation, Fixed night work, Shift work schedule, Occupations

## Abstract

**Background:**

The objective of this study was to examine the relationship between employee work schedule and failure in smoking cessation.

**Methods:**

Logistic regression was used to estimate the association between work schedule and failed smoking cessation rate among 4927 male workers who had attempted smoking cessation. The data was obtained from the annual Korean National Health and Nutrition Examination Survey from 2007 to 2015 (excluding data from 2013). An adjusted model, including demographic and occupational variables, was constructed after stratifying the data into two subgroups by age (the 19- to 40-year-old group and the 41- to 60-year-old group).

**Results:**

The percentage of smoking-cessation failure varied according to work schedule and age. The failure rate in the 19- to 40-year-old group was generally higher for all work schedule categories than in the 41- to 60-year-old group. In particular, the highest percentage (90.9%) of smoking-cessation failure was in the fixed overnight work group. After adjusting for demographic characteristics and work organization variables, the odds ratio for failed smoking cessation across all ages was 3.30 (95%CI 2.23–4.86) among the fixed overnight workers compared to the daytime workers. Both of the age-stratified subgroups maintained this relationship, with a notably higher OR in the 19- to 40-year-old group (OR 3.74, 95% CI 1.80–7.77).

**Conclusions:**

Fixed overnight work is likely to negatively affect smoking cessation compared to other work schedules. Tailored anti-smoking intervention programs are required based on work schedule.

## Background

Although smoking prevalence has been declining during the recent decades [1], it is still high in Korea, with an estimated 42.1% of South Koreans being smokers in 2013 [2]. This plays a major role in respiratory diseases, such as lung cancer, chronic obstructive pulmonary disease, and bronchitis, as well as cardiovascular diseases such as hypertension, coronary artery disease, and angina [1, 3]. Smoking cessation policies have been actively implemented to reduce smoking rates and have been found to be more cost effective than related health care services [4]. However, considering that the majority of the world population works outside the home, there should be a constant emphasis on smoking cessation in the workplace. Particularly, the fact that the smoking rate is reported to be highest in nighttime workers [5] demonstrates that professionals, such as occupational physicians or health care providers, should offer smoking intervention programs focused on night workers.

Night work is likely to increase the incidence rate of various diseases, including digestive disorders, sleep disorders, hypertension, diabetes, dyslipidemia, atherosclerosis, and breast cancer, compared to day work [6–9]. The mechanisms of the diseases associated with night work are rather complex, though a general mechanism could be the disturbance of the circadian rhythm [10]. Recently the conclusion were made that night-shift work involving circadian disruption is probably carcinogenic to humans (group 2A) by the International Agency for Research on Cancer (IARC) [11]. Melatonin is a hormone that maintains the circadian rhythm and helps regulate other hormones such as female sex hormones. When melatonin, which is secreted mostly after sunset and decreases right before sunrise, is suppressed by artificial light while working at night, it results in disturbance of the circadian rhythm, as well as shortage of the hormone itself [12]. Sleep disorders, digestive problems, and decreased function and concentration due to disruption of the circadian rhythm are likely to increase an individual’s stress level, leading to maintained smoking use to release stress. However, studies drawing from working populations who attempted to quit smoking are rare. Specifically, very few studies have analyzed the relationships between factors that cause failure in smoking cessation and occupational backgrounds. One recent study focused on the rate of failed smoking cessation by occupation category, showing that young male workers engaged in service and sales are vulnerable in quitting smoking compared to those in other categories [13]. However, to our knowledge, the relationship between nighttime work and smoking-cessation failure has not yet been studied. Smoking cessation policies at workplaces include smoking cessation counselling by health managers, periodic smoking cessation meeting, sharing smoking cessation diary among the meeting members, group medication, anti-smoking education. These various smoking cessation policies are usually targeted toward the day workers as the health managers at workplaces who are in charge of the policies are mostly day workers. Night work includes both fixed overnight work and night-including rotating work, and the two work schedules are expected to be different in terms of life pattern and circadian rhythm adjustment [6, 14–16]. Though some previous studies that investigated the two work schedules reported no significant differences in adjustment or greater negative effects with rotating work than fixed overnight work [15], some studies have concluded that circadian adaptation is impossible even with consistent fixed overnight work [14, 16].

To this end, the present study instigated the following hypotheses: (1) the smoking-cessation failure rate in the overnight work group is higher compared to day work, and (2) different to night-including rotation work.

## Subjects and methods

### Study population

**Fig. 1:**
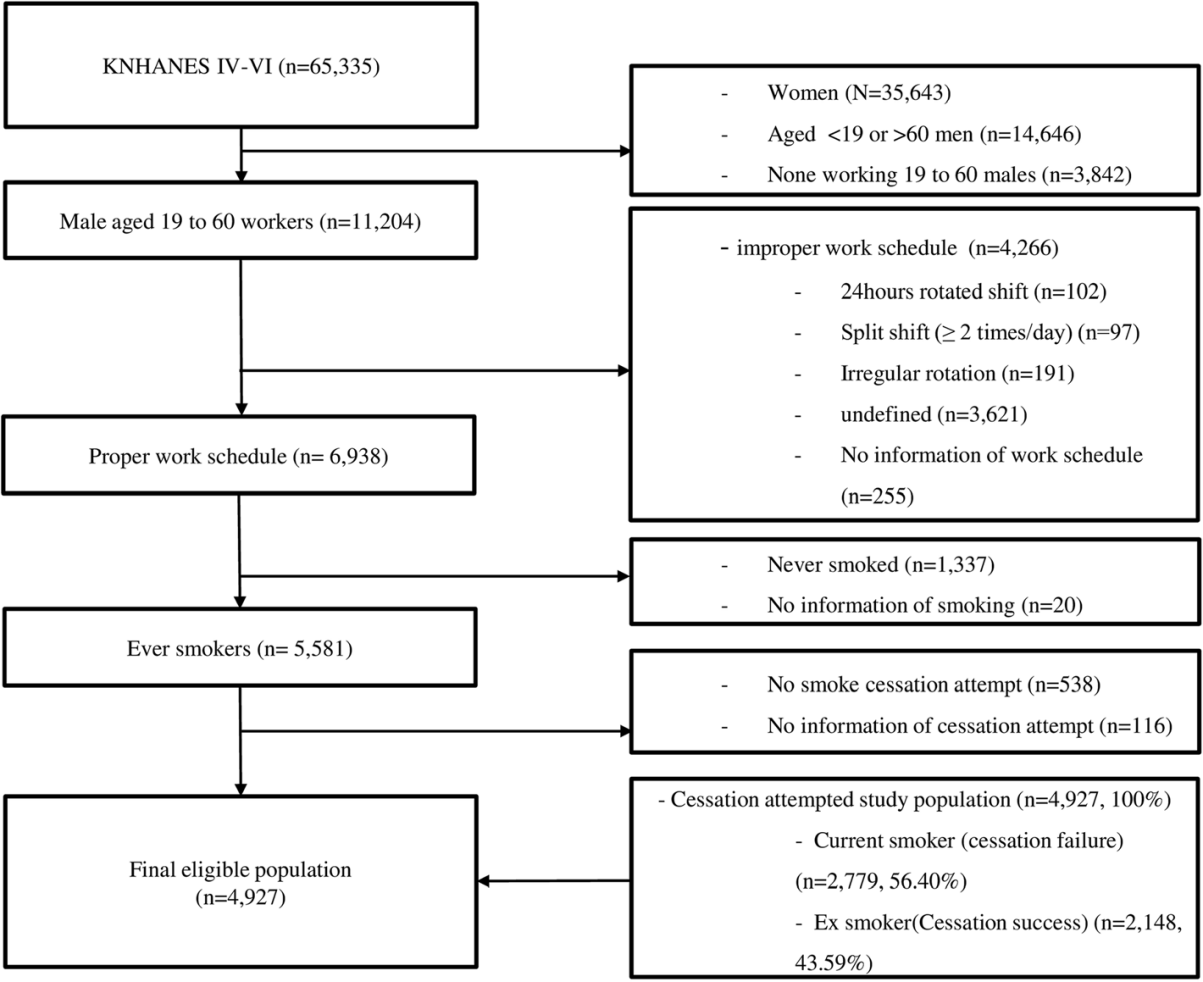
Explanation of the study population

The Korean National Health and Nutrition Examination Survey (KNHANES) is a national survey representing the non-institutionalized, civilian population in South Korea and is conducted annually by the Korean Ministry of Health and Welfare and the Korean Center for Disease Control and Prevention (KCDC). The characteristics of the data are similar to the National Health and Nutrition Examination Survey (NHANES) administered in the US. The data in the survey were collected by trained interviewers and examiners and comprise three sub-surveys: a health interview, a health examination, and a nutrition survey. The participants are selected randomly every year by computer algorithm from the samples which were stratified by cities, ages, sexes etc. Therefore it is less likely for the participants to be duplicated in the study. Further details of the KNHANES are available on the KCDC website (https://knhanes.cdc.go.kr/knhanes/eng/index.do) and in previously published literatures [13, 17]. Present study included the data from KNHANES IV and V that was collected from 2007 to 2015 (excluding 2013) and partial data from VI (2014 and 2015 only). We excluded the data from 2013 because the relevant information on smoking-cessation attempts was not obtained in that year. The total number of participants was 65,335 with a response rate of 78.3 to 80.8% throughout the phases. Among these participants, the following were excluded: all women (35,643), men less than 19 or more than 60 years old (14,646), non-working men age 19 to 60 (3842), men who did not satisfy our work schedule classifications of daytime shift, evening shift, fixed overnight shift, and night-including rotation shift (4266), men who were non-smokers (1337), men for whom there was no information regarding smoking or smoking-cessation attempts (136), and men who did not attempt smoking cessation (538). The remaining 4927 subjects who were 19- to 60-year-old male workers with a suitable work schedule and who had attempted smoking cessation were finally enrolled as the eligible population for the present study (Fig. 1).

### Measurements

All responses were self-reported. For the purpose of this study, current smokers were defined as individuals who smoked either daily or occasionally in the past month. Ex-smokers were defined as individuals who had smoked more than 100 cigarettes in the past but had quit for at least the last month. Failure in smoking cessation was defined as a current smoker who had attempted smoking cessation but resumed smoking. The demographic and working organization variables were included as control variables in the adjusted analysis.

The demographic variables included age, education level, economic status, marriage status, and perceived stress level and were defined as follows. Education level was divided into three levels of “middle school or less,” “high school,” and “university or more.” Economic status was determined by categorizing the family income into quartiles. For marital status, “married or cohabiting” was regarded as a “positive” marital status and “separated or not married” as a “negative” marital status. Perceived stress was categorized from the response to the question “How much stress do you feel on a daily basis?” using a four-point Likert point scale. The response of either “very much” or “much” was defined as “high” perceived stress, and “a little” and “little” was defined as “low” perceived stress.

The working condition variables included four categories of working schedule, weekly work hours, job category, and type of employment. The working schedule classification was determined from the question “Do you work during the day time (between 6 am and 6 pm) or at another time?” The individuals were allowed to choose from the following schedules: (1) day time, (2) evening, between 2 pm and 12 am, (3) night time, between 9 pm to 8 am, (4) night-included regular rotation shift, (5) 24-h shift, (6) split shift, more than 2 times a day, (7) irregular rotation shift, and (8) others. We excluded respondents from classifications 5 to 8 due to the small number and high uncertainty. The response “day time” was classified as “day time work,” “evening” as “evening time work,” “night time” as “fixed overnight work,” and “night-included regular rotation shift” as “night-included rotation work.” Work hours were defined as the average weekly working hours including overtime work and excluding meal times occurring during work. The nine job categories were classified into three subcategories: “administrator,” “professional,” and “clerk” as “office workers”; “service worker” and “salesperson” as “service and sales”; and “agricultural, forestry, and fishery workers,” “technicians,” “machinery operators,” and “laborers” as “manual work.” The type of employment was classified as permanent employment, temporary employment, daily work, and other (employer, self-employed, unpaid family workers, etc.)

### Statistical analyses

We generated descriptive statistics for the overall study population, as well as bivariate variables of the younger population (aged between 19 and 40) and of the older population (aged between 41 and 60). Student’s t-test, Rao-Scott Chi-square test, and survey logistic regression were performed. For multivariate analysis, multiple logistic regression analysis was conducted to estimate the odds ratio (OR) for failed smoking cessation among Korean male workers. First, an unadjusted model using only work schedule was estimated, then two adjusted models (model 1: work schedule and demographic variables, model 2: work schedule, demographic variables, and work organization variables) were estimated. The statistical analyses were performed using SAS, version 9.4 (Statistical Analysis System Institute, Cary, NC, USA). A *P*-value less than 0.05 was considered statistically significant.

## Results

**Fig. 2:**
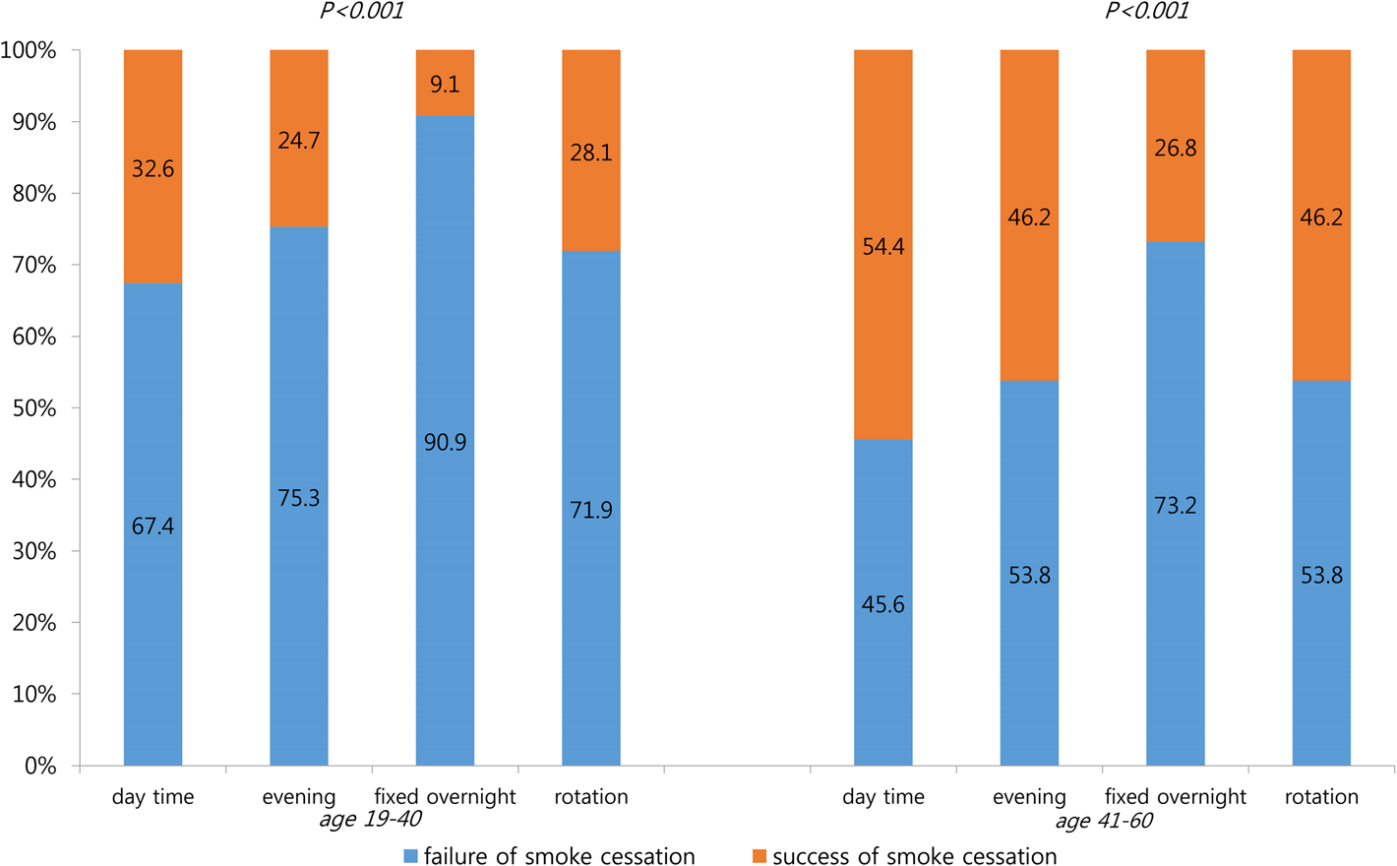
Age-specific percentage of smoking-cessation failure by work schedule, merged data from 2007 to 2012, 2014–2015

A total of 4927 workers were enrolled in the study. Figure 2 displays the age-specific percentage of failure in smoking cessation by work schedule, generally showing a higher failure risk among younger male workers, especially if they were fixed overnight workers (90.9%). The older group showed similar features of failure rates of smoking cessation regarding work schedule, with the highest failure rate in the fixed overnight work group.

A general description of the study population is displayed in Table 1. More than 80% of the population was day time workers. Fixed overnight workers and night-included rotation workers composed 4.0 and 5.1% of the population, respectively. The work organization characteristics revealed that the majority of the population (60.7%) was working more than 40 h a week, and that 52.8% of people were permanent workers. Office workers and service workers composed almost 58% of the population. The study population tended to be well educated (84.8%), economically stable (71.7%), living with a spouse (80.8%), and had a low level of perceived stress (69.8%). However, the characteristics were quite varied according to age as the younger male workers were more likely to be night workers, non-manual workers, or permanent workers; more educated; less likely to be married; and had more stress than the older male workers. The characteristics also varied according to work schedule (Table 2). Workers were more likely to be manual workers (59.3%), to work 40 h a week or less (64.8%), or be unmarried or separated from their spouse (31.6%) if they were engaged in fixed night work.

**Table 1:** Demographic and occupational characteristics of the subjects stratified by age

	Total		19- to 40-year-old men		41- to 60-year-old men		*p-value*
	N	%(SE)	N	%(SE)	N	%(SE)	
Age (mean (SD))	38.2 (0.1)		32.1 (0.1)		49.6 (0.1)		<0.01
Work schedule							
Day time (06 am-06 pm)	4119	83.6 (0.5)	1594	79.6 (0.9)	2525	86.4 (0.6)	
Evening time (02 pm-12 am)	361	7.3 (0.4)	190	9.5 (0.7)	171	5.9 (0.4)	
Fixed overnight (09 pm-08 am)	196	4.0 (0.3)	99	4.9 (0.5)	97	3.3 (0.3)	
Night-included regular rotation	251	5.1 (0.3)	121	6.0 (0.5)	130	4.4 (0.4)	<0.01
Job category							
Office work	2036	41.6 (0.7)	959	48.5 (1.1)	1077	37.0 (1.1)	
Service and sales work	824	16.9 (0.5)	392	19.8 (0.9)	432	14.8 (0.9)	
Manual work	2032	41.5 (0.7)	627	31.7 (1.0)	1405	48.2 (1.0)	<0.01
Type of employment							
Permanent	2603	52.8 (0.7)	1324	66.1 (1.1)	1279	43.8 (0.9)	
Temporary	297	6.0 (0.3)	176	8.8 (0.6)	121	4.1 (0.4)	
Daily workers	246	5.0 (0.3)	74	3.7 (0.4)	172	5.9 (0.4)	
^a^Other	1781	36.1 (0.7)	430	21.4 (0.9)	1351	46.2 (0.9)	<0.01
Work hour							
40 h per week or less	1938	39.3 (0.7)	742	37.0 (1.1)	1196	40.9 (0.9)	
More than 40 h per week	2989	60.7 (0.7)	1262	63.0 (1.1)	1727	59.1 (0.9)	0.01
Educational level							
Middle school or less	749	15.2 (0.5)	51	2.6 (0.4)	698	23.9 (0.8)	
High school	1926	39.1 (0.7)	814	40.6 (1.1)	1112	38.0 (0.9)	
University or more	2252	45.7 (0.7)	1139	56.8 (1.1)	1113	38.1 (0.9)	<0.01
Home income quartile							
Low	272	5.6 (0.3)	103	5.1 (0.5)	169	5.8 (0.4)	
Medium low	1115	22.8 (0.6)	499	25.0 (1.0)	616	21.3 (0.8)	
Medium high	1685	34.5 (0.7)	762	38.2 (1.1)	923	31.9 (0.9)	
High	1818	37.2 (0.7)	632	31.7 (1.0)	1186	41.0 (1.2)	0.01
Marriage status							
Positive	3981	80.8 (0.6)	1291	64.4 (1.1)	2690	92.0 (0.5)	
Negative	946	19.2 (0.6)	713	35.6 (1.1)	233	8.0 (0.5)	<0.01
Perceived stress							
Low	3441	69.8 (0.7)	1306	65.2 (1.1)	2135	73.0 (0.8)	
High	1486	30.2 (0.7)	698	34.8 (1.1)	788	27.0 (0.8)	<0.01

^a^includes employer, self-employed, unpaid family workers, etc.

**Table 2:** Demographic and occupational characteristics of the subjects according to work schedule

	Day time (06 am-06 pm)		Evening time (02 pm-12 am)		Fixed overnight (09 pm-08 am)		Night included regular rotation		*p-value*
	N	% (SE)	N	% (SE)	N	% (SE)	N	% (SE)	
Age (yr, mean (SD))	43.9 (0.2)		39.8 (0.6)		40.3 (0.8)		41.2 (0.6)		<0.01
Job category									
Office work	1826	44.6 (0.8)	139	38.8 (2.6)	31	16.0 (2.6)	40	16.0 (2.6)	<0.01
Service and sales work	596	14.6 (0.6)	126	35.2 (2.5)	48	24.7 (3.1)	54	21.6 (3.1)	
Manual work	1668	40.8 (0.8)	93	26.0 (2.3)	115	59.3 (3.5)	156	62.4 (3.5)	
Type of employment									
Permanent	2177	52.8 (0.8)	75	20.8 (2.1)	115	58.7 (3.5)	236	94.0 (1.5)	<0.01
Temporary	201	4.9 (0.3)	63	17.5 (2.0)	25	12.7 (2.4)	8	3.2 (1.1)	
Daily workers	226	5.5 (0.4)	9	2.5 (0.8)	9	4.6 (1.5)	2	0.8 (0.6)	
^a^Others	1515	36.8 (0.8)	214	59.3 (2.6)	47	24.0 (3.1)	5	2.0 (0.9)	
Work hour									
40 h per week or less	1544	37.5 (0.8)	192	53.2 (2.6)	127	64.8 (3.4)	75	29.9 (2.9)	<0.01
More than 40 h per week	2575	62.5 (0.8)	169	46.8 (2.6)	69	35.2 (3.4)	176	70.1 (2.9)	
Educational level									
Middle school or less	656	15.9 (0.6)	46	12.7 (1.8)	27	13.8 (2.5)	20	8.0 (1.7)	<0.01
High school	1510	36.7 (0.8)	151	41.8 (2.6)	120	61.2 (3.5)	145	57.7 (3.1)	
University or more	1953	47.4 (0.8)	164	45.4 (2.6)	49	25.0 (3.1)	86	34.3 (3.0)	
Home income quartile									
Low	226	5.5 (0.4)	25	7.0 (1.4)	14	7.2 (1.8)	7	2.8 (1.0)	0.12
Medium low	944	23.1 (0.7)	80	22.4 (2.2)	39	20.0 (2.9)	52	20.8 (2.6)	
Medium high	1383	33.8 (0.7)	121	33.9 (2.5)	76	39.0 (3.5)	105	42.0 (3.1)	
High	1535	37.6 (0.8)	131	36.7 (2.6)	66	33.8 (3.4)	86	34.4 (3.0)	
Marriage status									
Positive	3407	82.7 (0.6)	241	66.6 (2.5)	134	68.4 (3.3)	199	79.3 (2.6)	<0.01
Negative	712	17.3 (0.6)	120	33.6 (2.5)	62	31.6 (3.3)	52	20.7 (2.6)	
Perceived stress									
Low	2869	69.7 (0.7)	244	67.6 (2.5)	145	74.0 (3.1)	183	72.9 (2.8)	0.30
High	1250	30.3 (0.7)	117	32.4 (2.5)	51	26.0 (3.1)	68	27.1 (2.8)	

^a^includes employer, self-employed, unpaid family workers, etc.

As shown in Table 3, fixed overnight work was found to be strongly predictive of failure in smoking cessation in the unadjusted model (OR 3.90, 95% CI 2.70–5.77), the demographic-adjusted model (model 1, OR 3.39, 95% CI 2.30–4.96), and the demographic- and work organization-adjusted model (model 2, OR 3.30, 95% CI 2.23–4.86). Night-included rotation work was predictive of slightly higher failure rate in smoking cessation compared to daytime workers in the unadjusted model (OR 1.42, 95% CI 1.10–1.85) and the model 2 (OR 1.34, 95% CI 1.01–1.79). After age stratification into two groups, as seen in Table 4, fixed overnight work was predictive of high odds of failing to quit smoking in the younger male workers (OR 3.74, 95% CI 1.80–7.77). The odds ratio of failure in smoking cessation declined to 3.19 (95%1.99–5.11) in fixed overnight workers in their 40s or 50s. Night-included rotation work was predictive of an approximately 50% higher odds of failure in smoking cessation in older male workers compared to day time work (OR 1.51, 95% CI 1.03–2.20).

**Table 3:** The odds ratio of smoking-cessation failure by work schedule

	Unadjusted				Model 1				Model 2		
	OR	95%CI			OR	95%CI			OR	95%CI	
Work schedule											
Day time (06 am-06 pm)	1				1				1		
Evening time (02 pm-12 am)	1.59	(1.27	1.99)		1.31	(1.03	1.66)		1.26	(0.99	1.62)
Fixed overnight (09 pm-08 am)	3.90	(2.70	5.77)		3.39	(2.30	4.96)		3.30	(2.23	4.86)
Night-included regular rotation	1.42	(1.10	1.85)		1.24	(0.94	1.63)		1.34	(1.01	1.79)

Model 1: adjusted for demographic characteristics of age, education, household income, marital status, and perceived stress

Model 2: adjusted for demographic characteristics (model 1) and the work organization variables of weekly work hours, job category, and type of employment (permanent/temporary)

**Table 4:** Age stratified odds ratio of smoking-cessation failure by work schedule

					Age stratification						
	Total				19–40 years				41–60 years		
	OR	95%CI			OR	95%CI			OR	95%CI	
Work schedule											
Day time (06 am-06 pm)	1				1				1		
Evening time (02 pm-12 am)	1.26	(0.99	1.62)		1.23	(0.84	1.80)		1.37	(0.99	1.89)
Fixed overnight (09 pm-08 am)	3.30	(2.23	4.86)		3.74	(1.80	7.77)		3.19	(1.99	5.11)
Night included regular rotation	1.34	(1.01	1.79)		1.16	(0.75	1.80)		1.51	(1.03	2.20)

All models are adjusted for demographic characteristics of age, education, household income, marital status, and perceived stress and work organization variables of weekly work hours, job category, and type of employment (permanent/temporary)

## Discussion

In the present study, the association between failure in smoking cessation and work schedule was examined in 4927 male workers aged 19 to 60 years old who had attempted smoking cessation using data from the Korean National Health and Nutrition Examination survey collected from 2007 to 2015, excluding the 2013 survey data. Several existing studies have examined the effects of night work on smoking rates [6, 18, 19], but few studies of night work and smoking-cessation failure exist to our knowledge. We confirmed the hypothesis that men who work during the night would find it harder to quit smoking compared to day workers. Furthermore, we identified an interesting outcome in the differing intensity of this effect on fixed overnight work and night-included rotating work.

After detecting the greatest difference in smoking-cessation failure by age, specifically that the percentage of smoking-cessation failure was significantly higher in younger male workers across all types of work schedule (Fig. 2), an age-stratified analysis was performed. A similar pattern in the odds ratios (ORs) of smoking-cessation failure based on work schedule was identified in both of the age groups with the highest ORs for fixed overnight workers. However, the intensity of the effect of fixed overnight work was higher in the younger group. The different outcomes according to age could be explained by different perspectives on health issues among different age groups [13]. The elderly tend to have more fear about the possibility of health deterioration; moreover, their existing symptoms or illness regarding pulmonary disease or cardiovascular disease might affect smoking cessation in a positive way [20].

One of the assumptions regarding smoking-cessation failure in night workers is that the failure is related to sleep problems, and that night workers are vulnerable to sleep issues due to insufficient circadian adjustment [14]. Among smokers, smoking cessation is known to result in drowsiness while awake and sleep disturbances due to nicotine withdrawal [21], and maintaining nicotine serum levels reduces waking during sleep, thereby improving the quality of sleep [22]. In a normal circadian structure, melatonin is secreted at night due to its induction during the dark phase of the light-dark cycle. However, even a low intensity of light, such as a computer screen, is capable of interrupting melatonin secretion [23]. This desynchronization of the light-dark cycle manifests as various nonspecific clinical symptoms such as chronic fatigue and chronic sleep deprivation [24]. To avoid the sleepiness and difficulty concentrating derived from fixed fatigue and chronic sleep deprivation, night time workers require enhanced alertness, which can be obtained by smoking as it improves concentration, calculation skill, motor coordination, and alertness in the short term [25, 26]. The hypothesis ‘situations attention-demanding elicit smoking’ is in line with a previous article, which reveals certain conditions that are conducive to smoking [27] and to increased failure in attempting to quit.

The higher smoking cessation failure rate of night workers compared to the day workers can be explained by circadian rhythm and addiction. Many previous studies highlighted that the circadian disruption may increase the risk of substance abuse as circadian misalignment has a tendency to increase reward sensitivity and impulsivity [28–30]. Moreover, genes that control circadian rhythms are involved in regulating the dopaminergic reward circuitry and the regulation may be the one cause of addiction tendency [31, 32]. Compared to the day workers, the night workers could be more vulnerable to addiction due to the circadian misalignment, which can explain the higher failure rate of smoking cessation.

Another reason night workers are vulnerable to failure in quitting smoking is their disconnection from social relationships including family and friends. Due to opposite life patterns, overnight workers have little chance to maintain social relations, which markedly increases the possibility of social isolation. Family support is known to be one of the major factors leading to successful smoking cessation [33]. However, having a differing life pattern from the rest of the family can lessen the intensity of the family support due to the limited interaction. Also, difficulty in engaging in leisure activities due to an incompatible life pattern can lead to negative effects on mental health. The failure in maintaining social relations and leisure activities leads to continuous anxiety and stress, which increase the tendency for smokers to rely on nicotine’s effects [34, 35]. Nicotine is known to be a stimulant in low doses and a sedative in high doses [27]. During anxiety- and stress-provoking situations, the sedative effect of nicotine is desired to reduce muscle tension, stabilize the mood, and reduce aggression [27].

Night tasks, such as being a security guard, working in markets open 24 h, and food delivery service, tend to be monotonous. Moreover, the basic stimulation afforded in the day time is absent, increasing boredom and isolation. Therefore, the combination of monotonous night tasks and less stimulation causes intensive nicotine dependency during night work, making it hard to quit smoking.

An additional reason for the vulnerability of fixed overnight workers regarding smoking cessation is the possibility of limited access to health care resources. The company’s health managers whose job involves counseling smoke issues are usually day time workers, leaving work when the overnight workers attend the workplace. Night fixed workers may find it difficult to access the health care resources as smoking cessation support services are usually available during the daytime when fixed overnight workers are mostly sleeping. But on the other hand, it may be easier to access to the medical services as the workers can use their time during the day if they are willing to access the services as they are mostly free on daytime. There is still a lack of clear empirical research, which needs to be examined in the subsequent studies.

Despite the research-based background on smoking-cessation failure among night workers, the reason for the different OR between fixed overnight work and night-included rotation work was curious to us. Both of the work schedules include night time work, which is known for sleep deprivation, chronic fatigue, monotony, social insufficiency, and minimal leisure time. However, minimal attempts to describe the differences in health effects between fixed overnight work and night-included rotation work have been made. Moreover, the findings in the literature are rather inconsistent [14, 36]. Dirkx J et al. addressed fixed night work has less negative effect on circadian adjustment compared to the night rotation work [36]. However, according to one review article, the fixed night work is minimally effective in providing sufficient circadian adjustments; hence it cannot minimize the various health issues associated with night work [14]. Considering the two different views mentioned forward, workers’ nicotine dependence should either be higher in the night rotation group or similar between the fixed night workers and night included rotation workers. However, our results showed that smoke cessation failure was higher in fixed night work group. It could be assumed that fixed night work might be more vulnerable to circadian adjustment than night included rotation work due to the permanent (unless quitting the work) discrepancy between internal clock and light-dark cycle. This assumption requires further research.

Other hypotheses could shed light on this discrepancy. The OR of smoking cessation failure for lowest educated, lowest income, and unmarried participants was 1.23(95%CI 0.99–1.53), 1.34(0.99–1.81), and 1.22(95%CI 1.02–1.46) respectively. After adjusting factors including education, SES, and marital status, the OR of fixed overnight work group became smaller and the difference of the ORs would mean the combination effect of adjusted factors (Table 3). The adjusted OR for the fixed overnight group decreased from 3.90 to 3.30. (Table 3). The other work schedule group showed a decrease in OR as well. Therefore the lower education level, the lower SES, and the lower marital status could have affected higher smoking cessation failure in the working groups. In our study, the education level of the fixed night workers was significantly lower than of the night-included rotation group (*p = 0.001*, Table 2). As low socio-economic level, including education level, is a known factor for higher rates of smoking and lower smoking-cessation rates [37, 38], the different education distributions among the two groups could have resulted in the different smoking-cessation rates. Second, the percentage of married persons was lower in the fixed overnight work group than the night included rotation work group (Table 2). A previous meta-analysis identified that family support is a major positive factor associated with smoke cessation [33]. As less percentage of overnight workers have chance to obtain family (spouse) support than rotating shift workers, the overnight workers are likely to be vulnerable to smoke cessation. Third, social insufficiency is generally greater in fixed night workers, as night rotation shift workers have several days of a normal life pattern during the rotating schedule. The chronic stress derived from severe social insufficiency could result in a strong dependency on nicotine [39]. We assumed that the social insufficiency could have influenced especially on the younger worker’s higher smoking cessation failure. Comparing with the older adults, younger adults tend to participate actively in social activity favoring many peripheral social partners [40]. Maintaining the peripheral social relationships usually requires increasing time and energy [41]. Older adults tend to emphasize as many emotionally close relationships less focusing on the peripheral ones [40]. Younger rotation workers have a chance to participate in social network as their work schedule includes day work as well as night work. They can afford several evenings in a month to participate in social network constructing and maintaining their peripheral ties. However, fixed night workers seldom have a chance to participate in social network in weekdays as their work schedule pattern is opposite with majority of the workers who work every day times. The different work pattern with day time workers would have made social isolation which is studied to increase the nicotine dependency [39]. However, further studies regarding the age effect on smoke cessation among night included workers are required. Attention toward the health deterioration caused by rotation work has recently increased along with the conclusion from the International Agency for Research on Cancer (IARC) that night-shift work involving circadian disruption is probably carcinogenic to humans (group 2A) [11]. An awareness of the health impact of rotation shift work among workers could influence their tendency to focus on health issues, which might result in a higher willingness to attempt smoking cessation. However, less focus is made on the health impact of fixed night work, which involves night work, with the resulting circadian disruption. Therefore, willingness to maintain smoking cessation could be lower in fixed overnight workers compared to rotating workers despite the fact that it is likely to be even more harmful based on several published articles, as well as the present study [14, 42]. Finally, chronic fatigue, sleep deprivation, monotonous night tasks, family conflicts, social insufficiency, and minimal leisure time all increase stress, which would require the sedative effects of nicotine to relax the body and stabilize the mood. Furthermore, due to the chronic fatigue, sleep deprivation, and monotony associated with night tasks, additional alertness is required to perform tasks and could be produced by the stimulant effects of nicotine delivered through smoking. It is assumed that both sedative effect and stimulant effect are required to the overnight workers as they are exposed both to the increased stress as well as the sleep deprivation and monotony. However, further studies are needed to identify whether sedative nicotine effect or stimulant effect is more desired among the overnight workers.

The present study faces several notable limitations. The major limitation lies in the cross-sectional study design due to the limitation of the data. We could not investigate the potential effects of a change in work schedule among study subjects. However, we analyzed the coincidence of current occupation category and the longest lifetime occupation category, which showed a 71.7% coincidence. Further prospective studies are required to confirm the association between smoking-cessation failure and work schedule. Second, due to the limitation of the data, we could not access the exact time of smoking cessation, which might have preceded the independent variable (work schedule) for some participants. However, we expect that an individual’s work schedule would usually be maintained and not change often. The high coincidence of the current job category and longest lifetime job category mentioned above also indirectly supports the low possibility of a change in work schedule. Furthermore, smoking cessation can be defined not as an unchangeable status after the achievement, but as maintaining the cessation status. To this point, even if an individual had stopped smoking before he started the current work, maintaining the cessation status at the present point implies the proper order between the independent variable (work schedule) and the outcome variable (smoking-cessation failure). The other limitation is that only male workers were involved in the study. Women in South Korea tend to hide their smoking habit due to the negative social view of women smoking. Hence, obtaining reliable information from the interview data was difficult. Therefore, we excluded women from the analysis to maintain accuracy.

Despite these limitations, the present study has several strengths. It confirmed the hypothesis that fixed overnight workers are vulnerable to failure in smoking cessation, which is the first such report, to our knowledge. Furthermore, we attempted to deduce the reasons for the different ORs between fixed overnight workers and night-included rotation workers based on previous literature. Additionally, the subjects in the study are representative of the non-institutionalized, adult, Korean, civilian population. Finally, we focused on the outcome of smoking cessation among the economically active male population; therefore, the results could be applied as basic information in workplace health-promotion programs.

There are several suggestions that could alleviate the circadian disruption and thus lower the smoking cessation failure rate among the night workers. The recent literatures highlighted that timed exercise is a zeitgeber for the human circadian system so that potential exercise-zeitgeber action could be used as one of the keys to alleviate the circadian disruption [43, 44]. Another study reported that the meal timing regulated the circadian rhythm in humans so that timed meal could help improve the circadian system disorders for night workers [45]. In addition, timed exercise or timed meal may alleviate monotony, social disconnection as well as increase health awareness. Thus, these interventions would eventually influence positively on quitting smoking for the night shift workers.

## Conclusions

The present study provides evidence that failed smoking cessation is related to work schedule, and that fixed overnight workers are the most vulnerable population. The different effects on smoking cessation from fixed overnight work and night included rotation work were discussed with several objective pieces of evidence; however, more studies with objective methods are needed to confirm the different effects between the two working groups. Our findings suggest that smoking intervention guidelines should be tailored according to the type of work schedule, particularly focusing on night work groups.

## Data Availability

All the data generated or analyzed during the study are included in this published article. The raw data of this research can be obtained on the KCDC website (https://knhanes.cdc.go.kr/knhanes/eng/index.do).

## Abbreviations

IARC: International Agency for Research on Cancer
KCDC: Korean Center for Disease Control and Prevention
KNHANES: The Korean National Health and Nutrition Examination Survey
NHANES: National Health and Nutrition Examination Survey
ORs: odds ratios
